# Supplementary material for: The utility of network analysis in the context of Indigenous Australian oral health literacy
Source: PLoS One. 2020 Jun 3;15(6):e0233972. doi: 10.1371/journal.pone.0233972 (PMC7269264; doi:10.1371/journal.pone.0233972)
Supplement: S1 Appendix — (PDF) [file pone.0233972.s001.pdf]

Health Literacy in Dentistry scale – Short Version (HeLD-14)

Ju X, Brennan DS, Parker E, Chrisopoulos S, Jamieson L. Confirmatory factor analysis of the health literacy in dentistry scale (HeLD) in the Australian population. Community Dent Health. 2018;35(3):140-147

Communication

Are you able to look for a second opinion about your dental health from a dental health professional?

Are you able to use information from a dentist to make decisions about your dental health?

Access

Do you know how to get a dentist appointment?

Do you know what to do to get a dentist appointment?

Receptivity

Are you able to pay attention to your dental or oral health?

Are you able to make time for things that are good for your dental or oral health?

Understanding

Are you able to read written information eg leaflets given to you by your dentist?

Are you able to read dental or oral health information brochures left in dental clinics and waiting rooms?

Utilisation

Are you able to carry out instructions that a dentist gives you?

Are you able to use advice from a dentist to make decisions about your dental health?

Support

Are you able to take family or a friend with you to a dental appointment?

Are you able to ask someone to go with you to a dental appointment?

Economic Barriers

Are you able to pay to see a dentist?

Are you able to pay for medication to manage your dental or oral health?
