# Supplementary figures and images for: The utility of network analysis in the context of Indigenous Australian oral health literacy
Source: PLoS One. 2020 Jun 3;15(6):e0233972. doi: 10.1371/journal.pone.0233972 (PMC7269264; doi:10.1371/journal.pone.0233972)

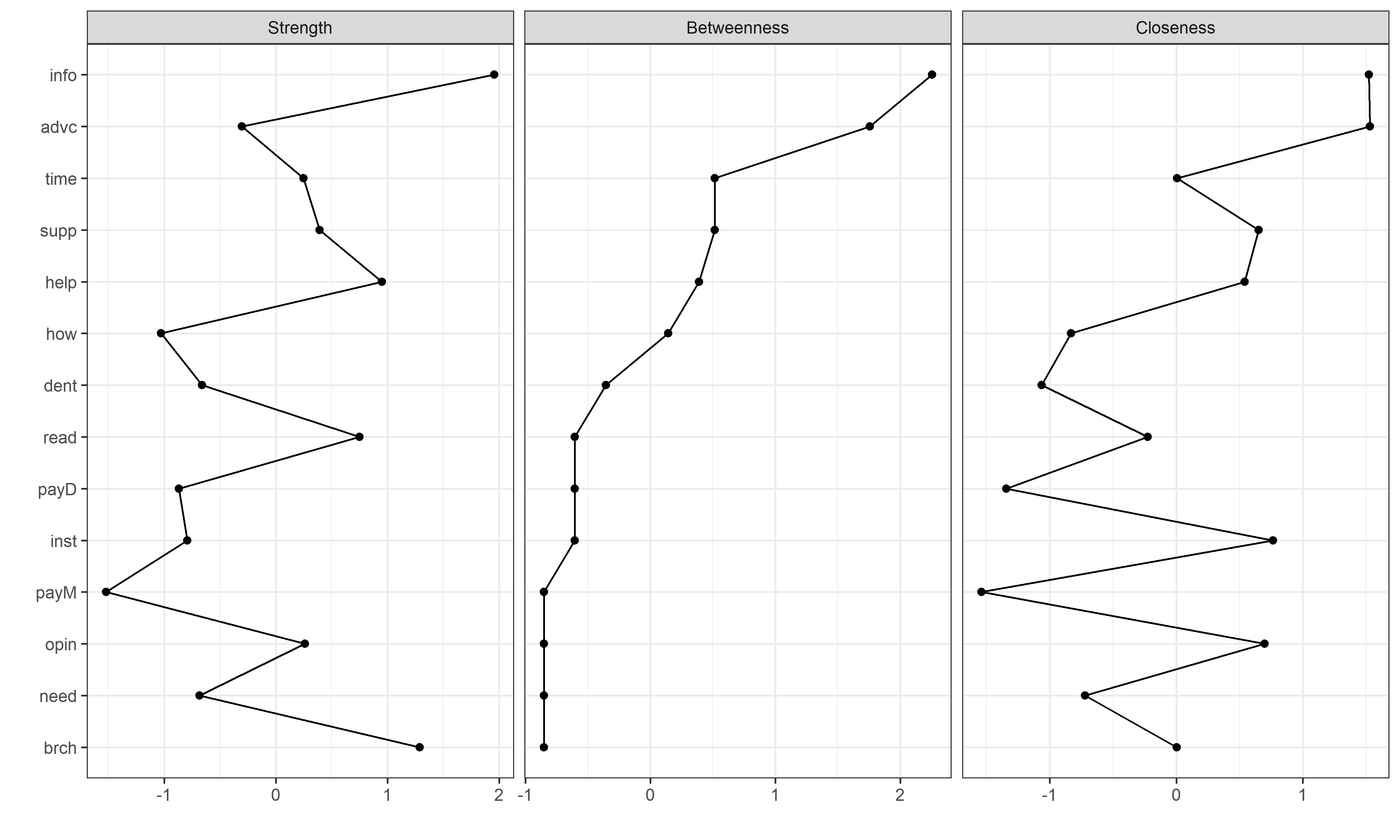

Supplement: S1 Fig — (TIFF) [file pone.0233972.s003.tiff]

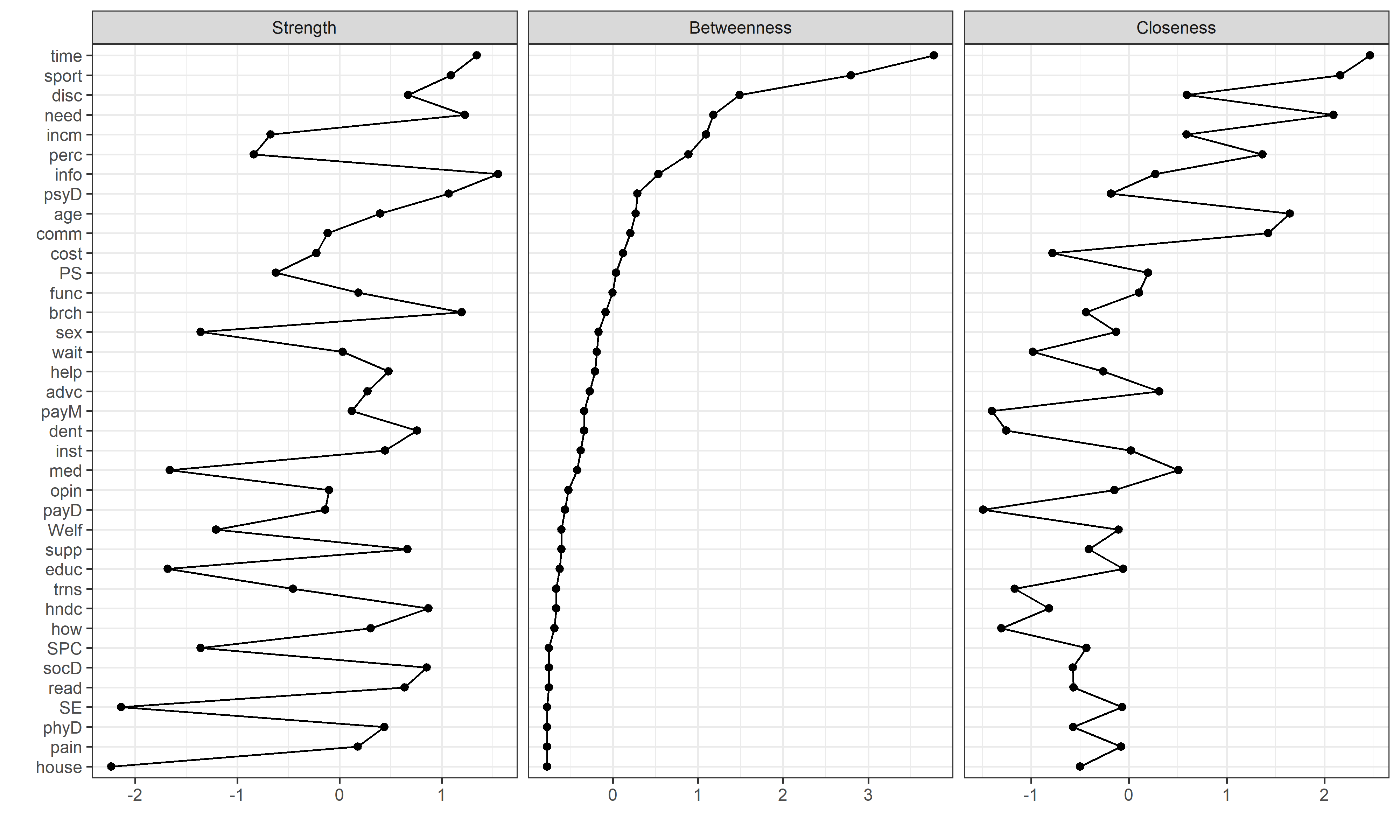

Supplement: S2 Fig — (TIFF) [file pone.0233972.s004.tiff]

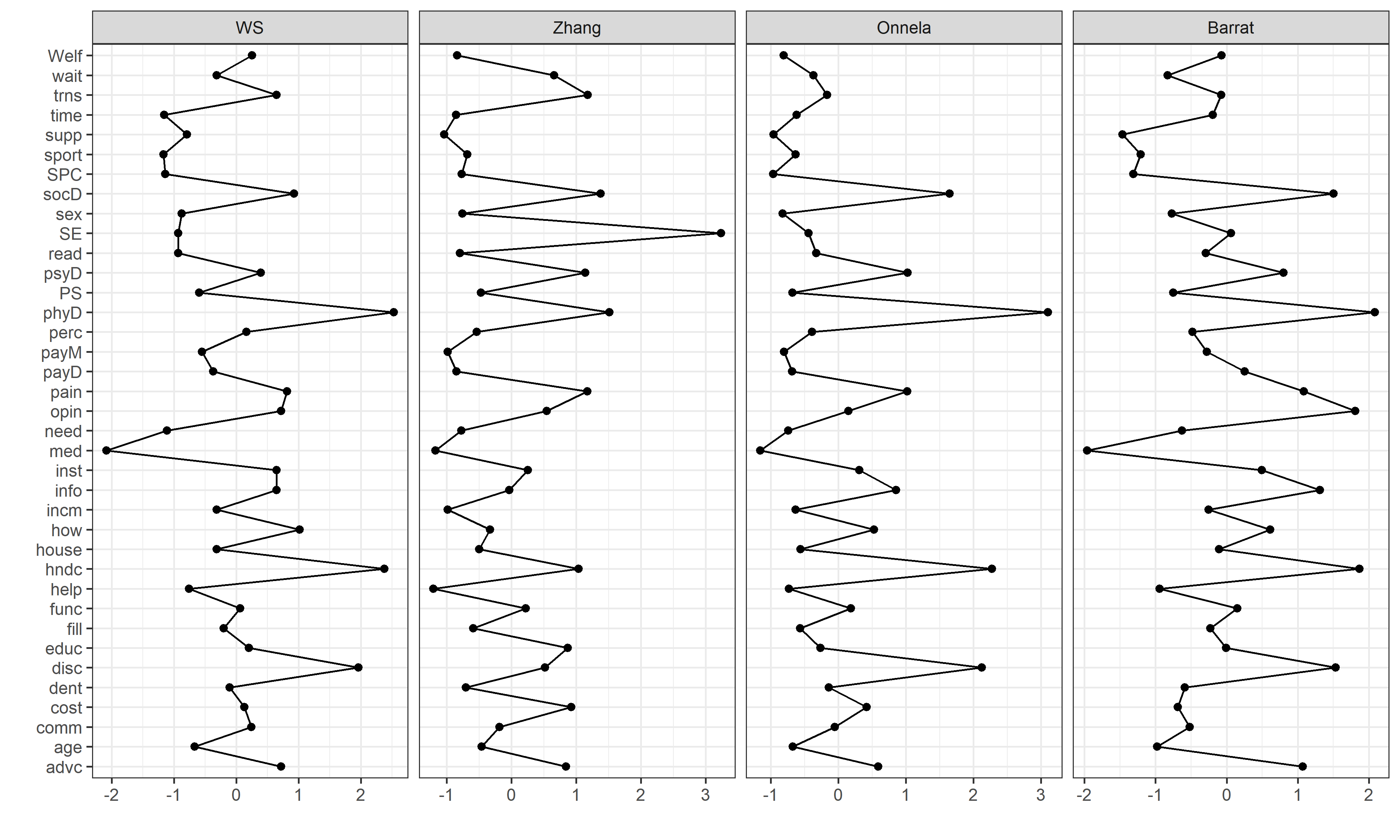

Supplement: S3 Fig — (TIFF) [file pone.0233972.s005.tiff]

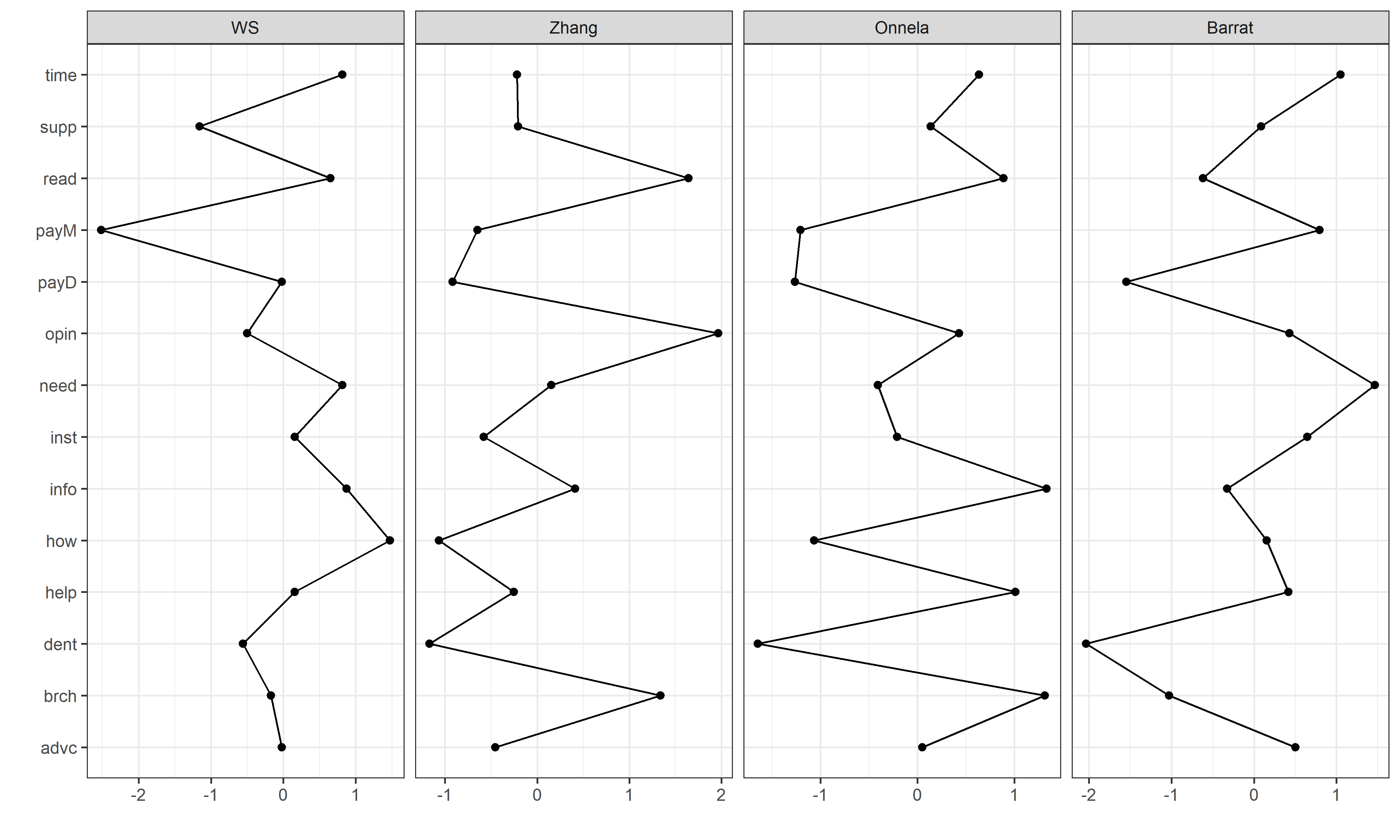

Supplement: S4 Fig — (TIFF) [file pone.0233972.s006.tiff]

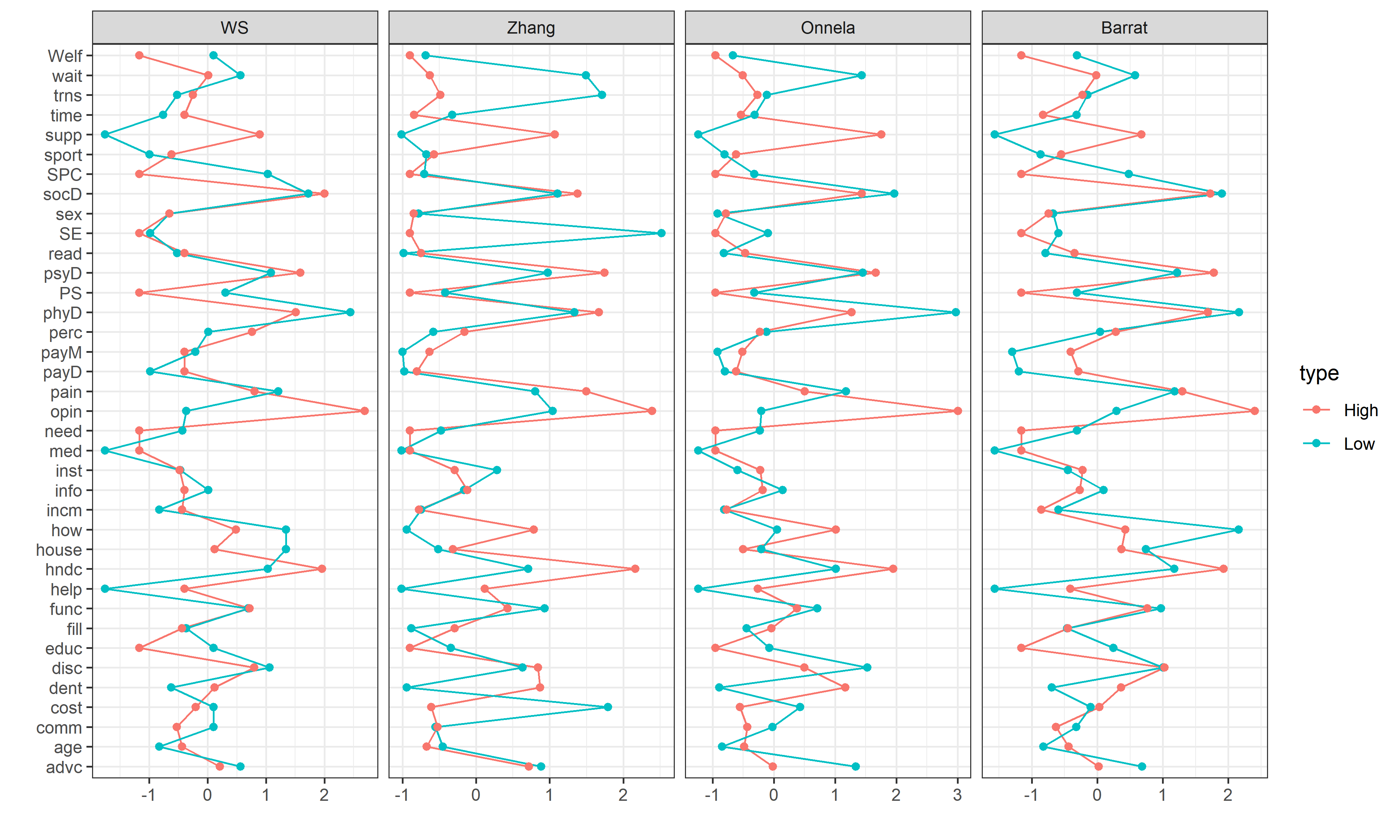

Supplement: S5 Fig — (TIFF) [file pone.0233972.s007.tiff]

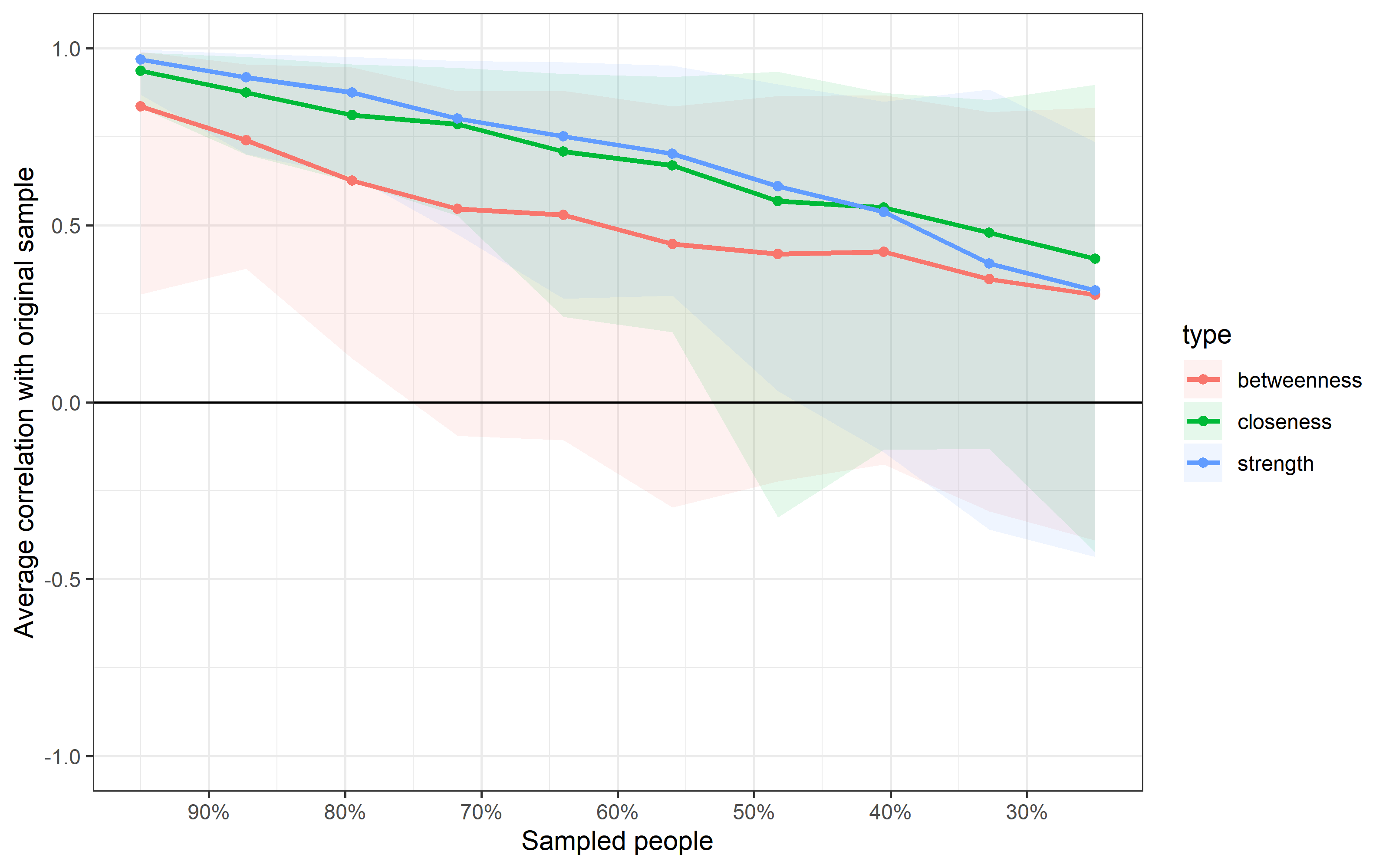

Supplement: S6 Fig — Lines indicate the average correlations between centrality indices of networks sampled with persons dropped and the original sample. Areas indicate the 95% confidence interval. (TIFF) [file pone.0233972.s008.tiff]

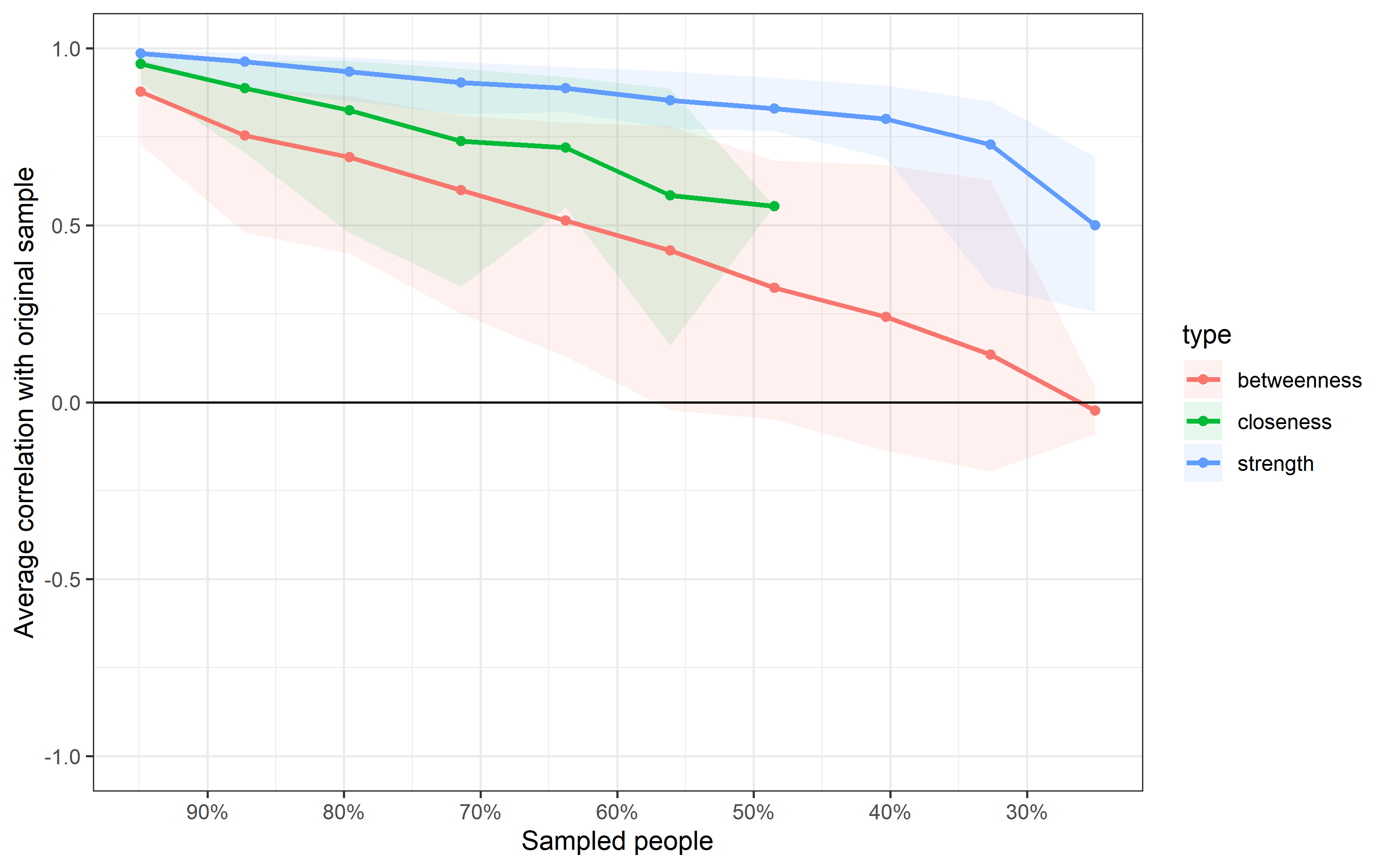

Supplement: S7 Fig — Lines indicate the average correlations between centrality indices of networks sampled with persons dropped and the original sample. Areas indicate the 95% confidence interval. (TIFF) [file pone.0233972.s009.tiff]

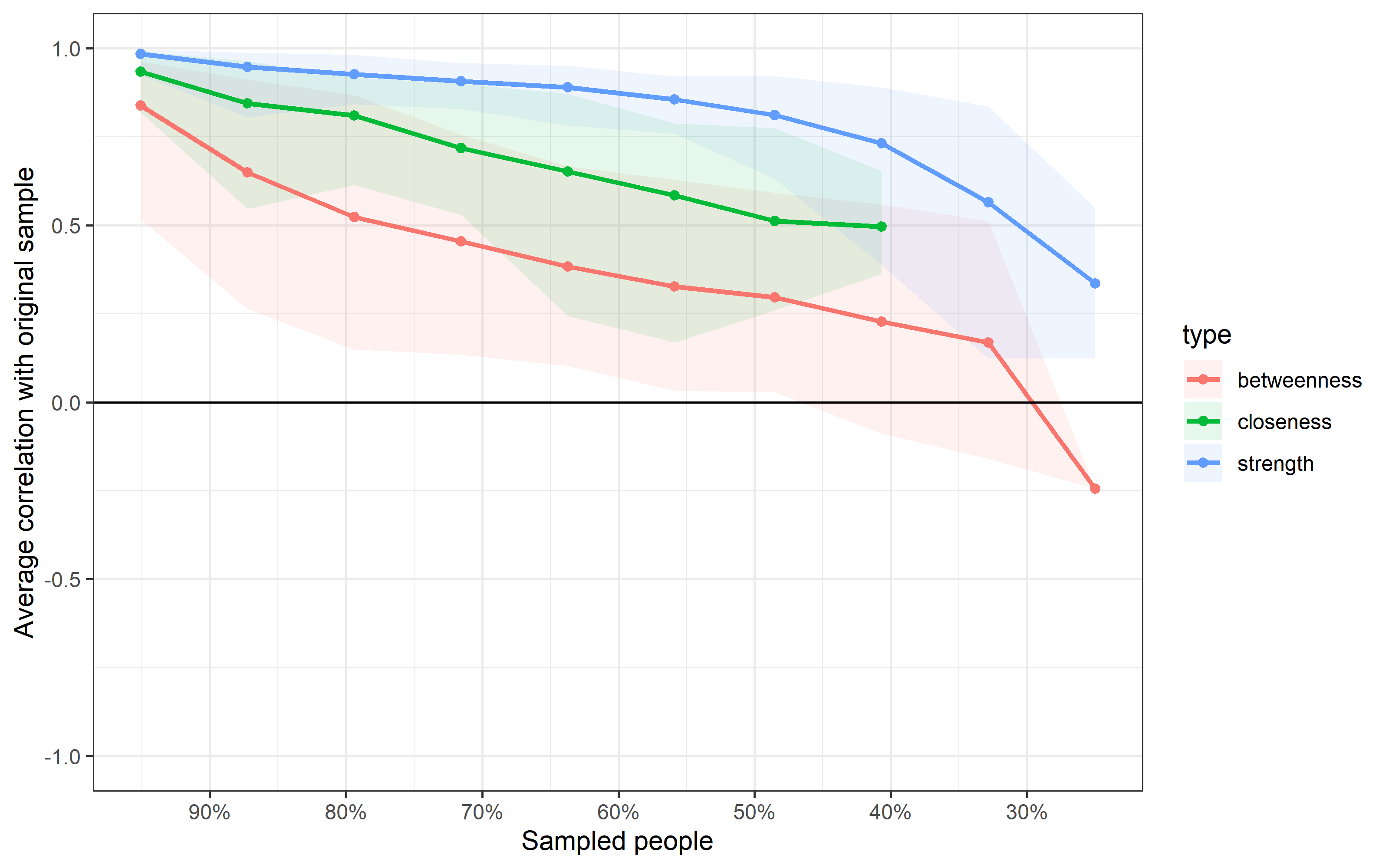

Supplement: S8 Fig — Lines indicate the average correlations between centrality indices of networks sampled with persons dropped and the original sample. Areas indicate the 95% confidence interval. (TIFF) [file pone.0233972.s010.tiff]

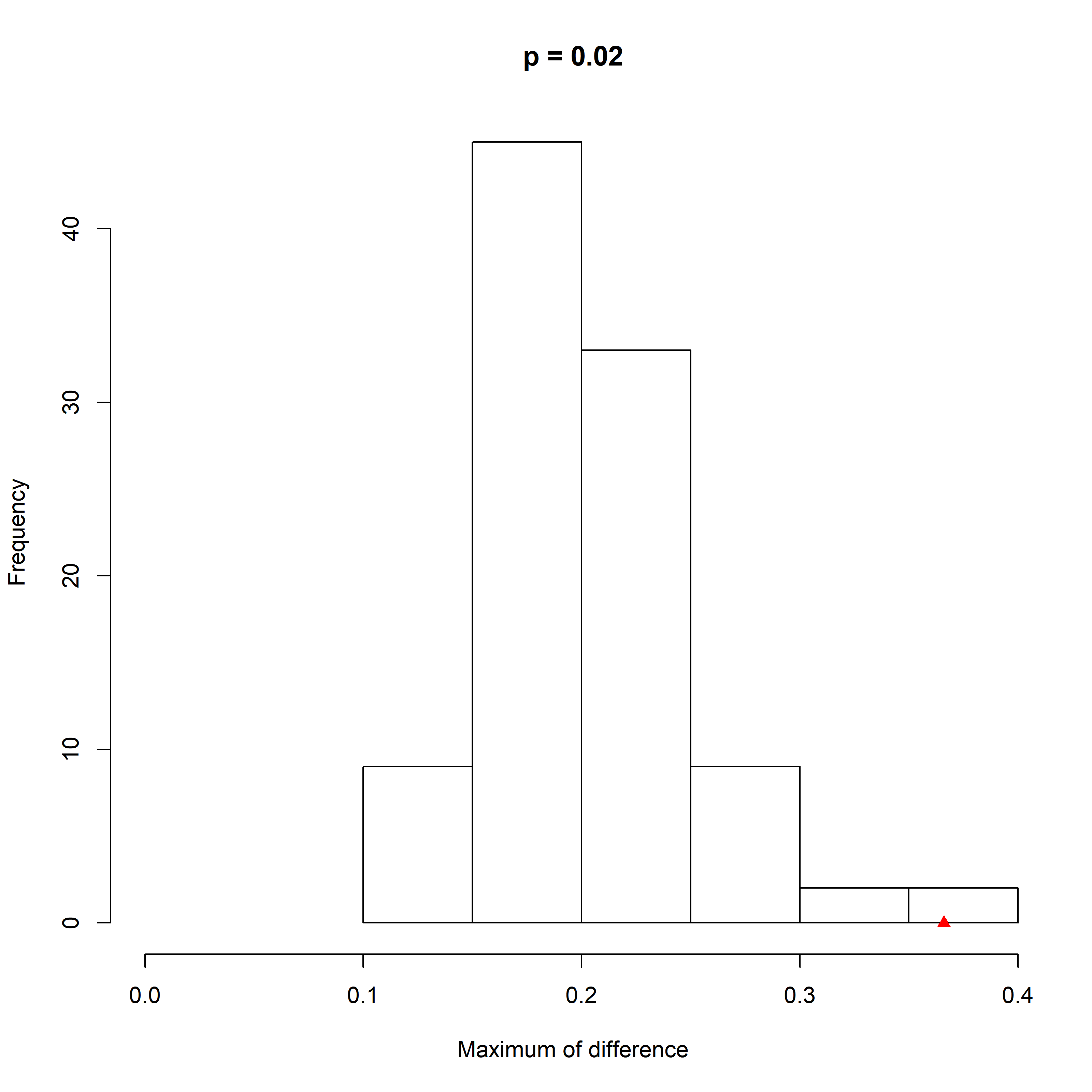

Supplement: S9 Fig — Red triangle indicates the observed difference. (TIFF) [file pone.0233972.s011.tiff]

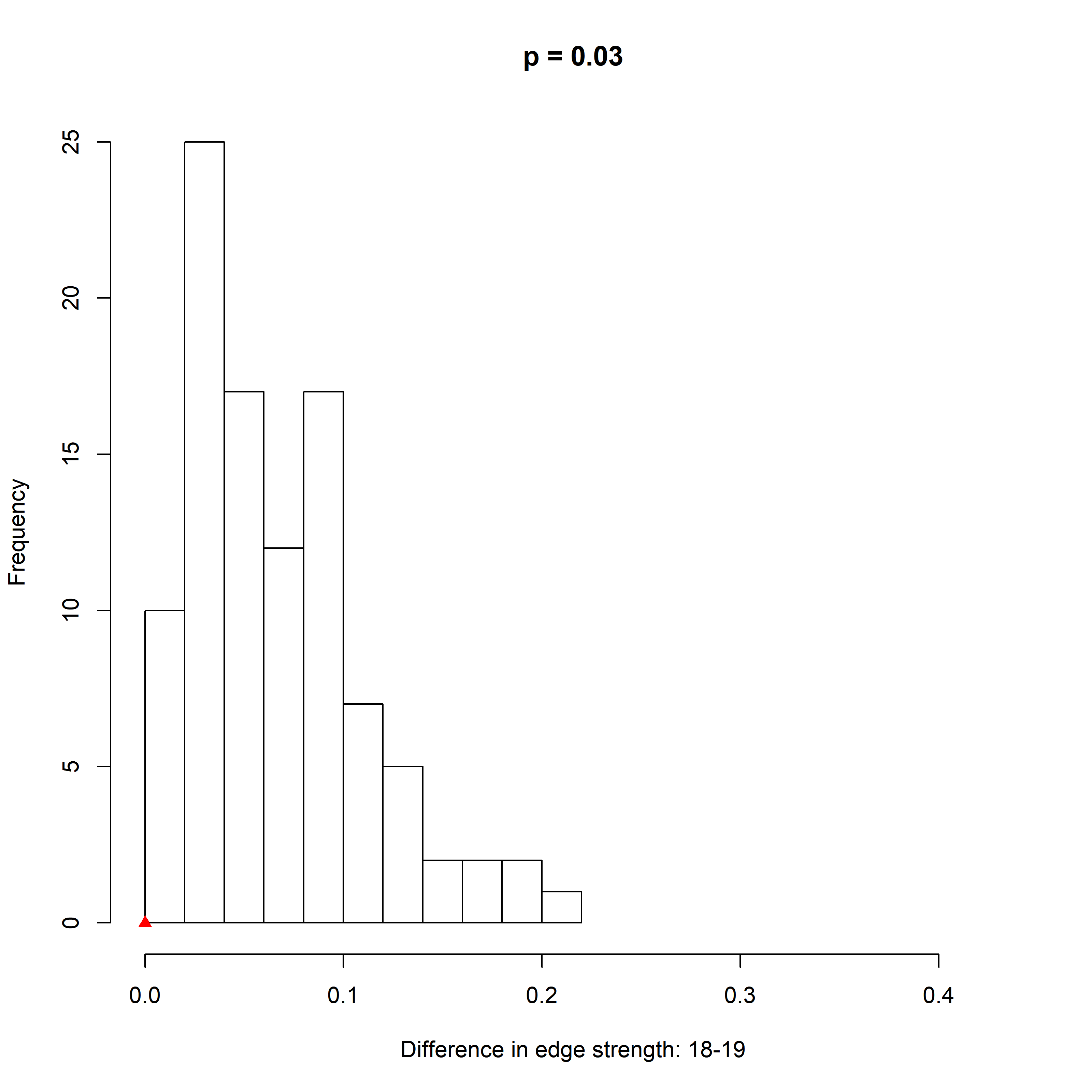

Supplement: S10 Fig — Red triangle indicates the observed difference. (TIFF) [file pone.0233972.s012.tiff]

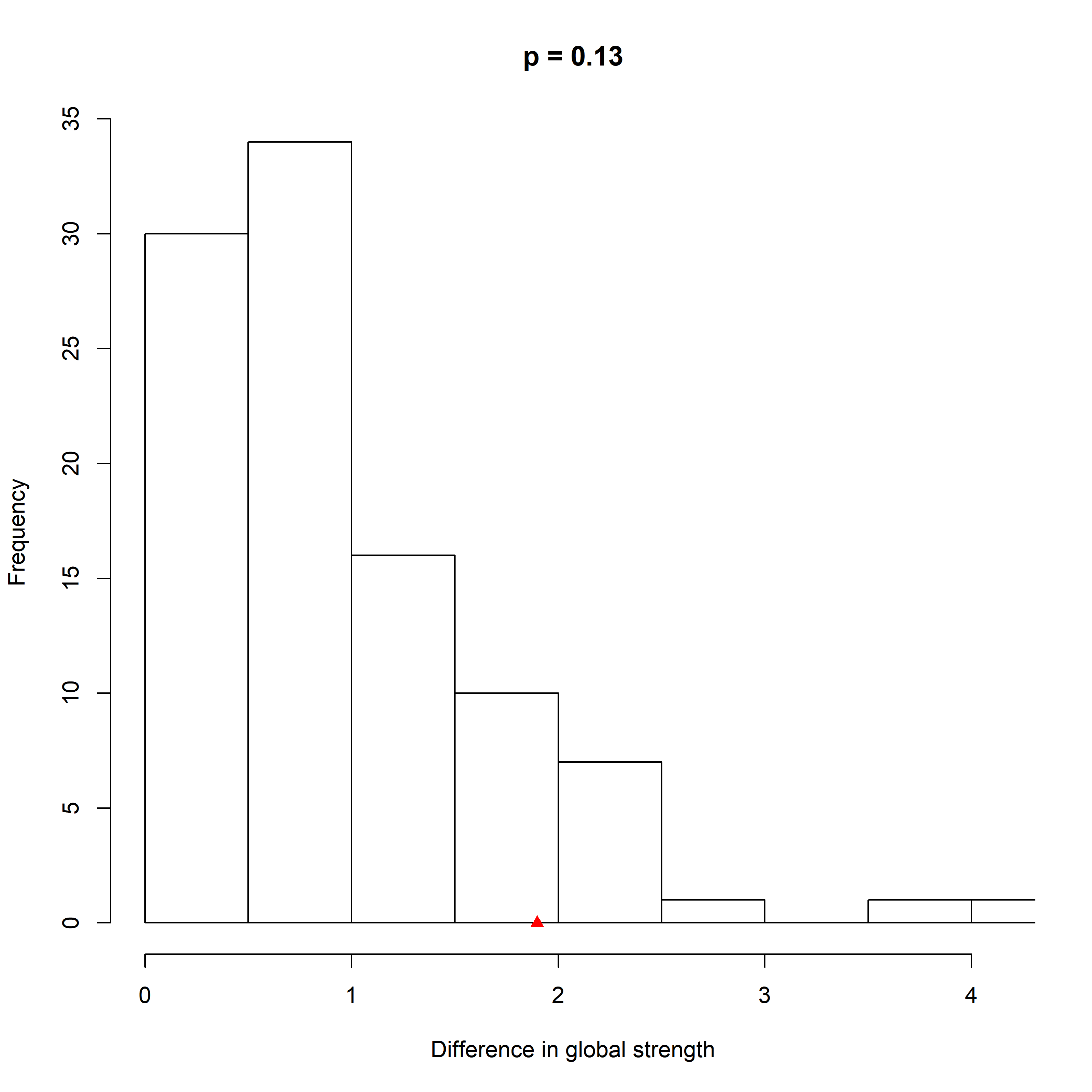

Supplement: S11 Fig — Red triangle indicates the observed difference. (TIFF) [file pone.0233972.s013.tiff]

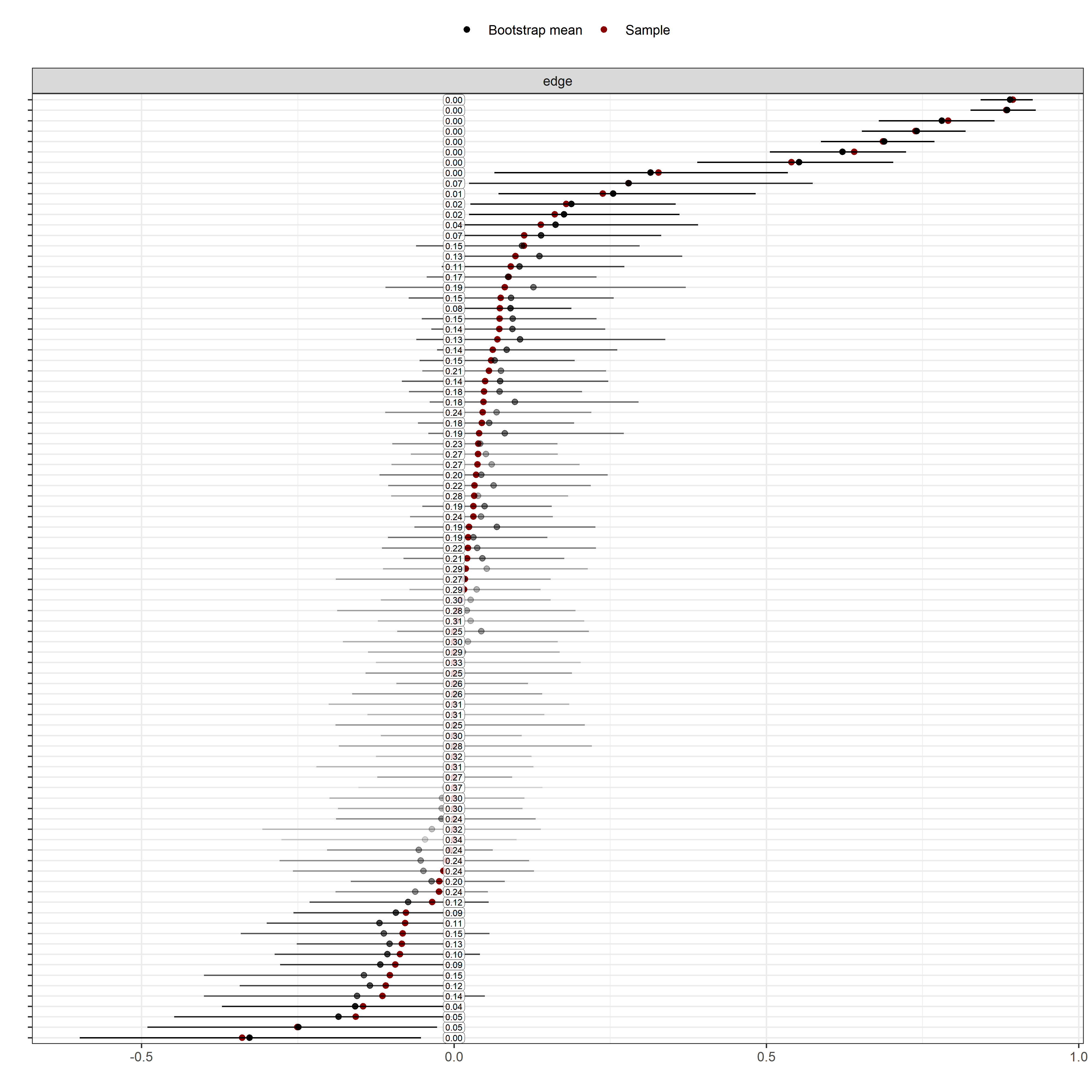

Supplement: S13 Fig — (TIFF) [file pone.0233972.s015.tiff]
