# Supplementary material for: The utility of network analysis in the context of Indigenous Australian oral health literacy
Source: PLoS One. 2020 Jun 3;15(6):e0233972. doi: 10.1371/journal.pone.0233972 (PMC7269264; doi:10.1371/journal.pone.0233972)
Supplement: Attachment — Submitted filename: Comments.docx [file pone.0233972.s018.docx]

Thank you for this opportunity to review this interesting paper using a novel analytical approach – network analysis. Findings from this study added new knowledge about the relationships between oral health literacy, personal and environmental factors. I provide the following comments for the authors’ consideration:

Abstract

Page 2 Methods: “… was used to estimate four regularised partial correlation networks”. Could you please explain these four networks briefly here?

Page 2 Results: “Oral health-related self-efficacy, sporting activities, and perceived oral health were the strongest positively associated nodes …” As for “perceived oral health”, do you mean “perceived oral health status?”

Introduction

Page 4: For the paragraph “Indigenous peoples worldwide are affected …”, it looks like some important rationale information is missing here. Why indigenous people have a disproportionate burden of oral health conditions? What is the status of oral health literacy in Indigenous people? I would suggest the authors add some justifications focusing on OHL and its relevant factors (e.g., self-efficacy, perceived stress) that were examined later in the present study, then introduce “network analysis”.

Methods

Page 4 Data: I suggest the authors briefly introduce the sampling method and recruitment strategies like how many communities were involved. It is much easier for readers to get an overall picture of the data collection process.

Page 5 Variables: How did the authors select these variables? Based on some theoretical framework? It is better to justify the rationale of selecting these OHL related factors, as this is closely related to next-step network analysis.

Page 5 Variables: Please briefly introduce the 7 conceptual dimensions of the OHIP-14. Does it mean higher scores indicate a higher quality of life for OHIP-14? Does it mean higher scores indicate higher control of the Sense of Personal Control scale? Does it mean higher scores indicate higher stress of PSS-14? Have the authors tested the reliability and validity of all relevant scales in the sample?

Page 6 Variables: “Barriers to the access of dental care included financial cost … Sociodemographic data included sex, age, level of formal education, income, welfare benefits, and household size (number of people per household).” Please make the measurement of these variables consistent and specific. For example, barriers of financial cost (yes/no?), sex (male/female?), age (continuous?) …

Page 6 Network estimation: I am wondering the sample size requirement for this network anlaysis. Is there some justification for the sample size and the number of nodes and edges?

Results

Page 11 Networks inference: The authors found that “the strongest negative connections emerged between items of Communication and Understanding (-0.25 and -0.34).” This finding looks a bit weird. Does it mean people with more frequent communication are less likely to understand oral health information?

Discussion

Overall, given health literacy (including OHL) is a context-and-content-specific term and network analysis highlights the role of networks and environments, I would suggest the authors add some discussions of the importance of health literacy environment, as recommended by the Australian Commission on Safety and Quality in Health Care. In addition, compared with the general population, there may be some specific and unique nature of networks within Indigenous communities for oral health. It would be much clearer to discuss the present findings within the Australian indigenous context, current dental health status and some implementation challenges for interventions.

Page 16: “In this study, the low OHL network presented greater density of node connectivity in comparison with the high OHL network.” What does this mean in the real world for oral health interventions? It would be better to discuss this finding within the oral health context, for example, is there any example of successful interventions for this densely connected networks?

Page 17: As for the inverse association between OHL and perceived stress (i.e., “… perceived stress was inversely associated. ”), is this finding consistent with previous research findings? What is the underlying reason or rationale?

Page 17: “Another study has shown a negative association between sense of personal control and perception of oral health [46].” Does this mean individuals with high sense of personal control are more likely to perceive poor oral health? Is this unexpected? What is the reason?

Page 18: As for the paragraph “Stable centrality measures can be helpful to understand how changes occur throughout the network structure, as ...” I would suggest the authors to interpret this finding with a specific example or intervention within the oral health context.

Page 18: “These results suggest that particularly items “use information” and “make time for dental health” comprise the core of the construct of oral health literacy.” What does this finding indicate for Australian indeginous people? Any implications?

Page 19: As for limitations of the present study, is there any limitation of this novel analytical approach – network analysis? For example, the uncertainty of bi-directional relationships between two nodes; unexpected results and complex interpretation.
