# Supplementary material for: The utility of network analysis in the context of Indigenous Australian oral health literacy
Source: PLoS One. 2020 Jun 3;15(6):e0233972. doi: 10.1371/journal.pone.0233972 (PMC7269264; doi:10.1371/journal.pone.0233972)
Supplement: Attachment — Submitted filename: Comments.docx [file pone.0233972.s020.docx]

Thanks for the authors’ updates and revision. The paper is much improved. Some minor comments are attached below:

Page 13 Line 233-239: As for 3.1 Results, I would suggest the authors add a table to describe the sample’s characteristics (e.g., sex, age, level of formal education, income, welfare benefits, household size, oral health literacy, other associated factors, and oral health outcomes), so that readers can better understand the findings for the context and the target population.

Page 19 line 370-384: Thanks for the authors’ detailed information on the Australian indigenous context. This context is helpful, but I cannot see how the current study’s findings are interpreted within this context. Are your findings aligned with previous literature you cited? Please discuss based on your findings.
