# Supplementary material for: The utility of network analysis in the context of Indigenous Australian oral health literacy
Source: PLoS One. 2020 Jun 3;15(6):e0233972. doi: 10.1371/journal.pone.0233972 (PMC7269264; doi:10.1371/journal.pone.0233972)
Supplement: Attachment — Submitted filename: Response to reviewers 15-04.docx [file pone.0233972.s021.docx]

April 15, 2020

**Response to reviewers**

We would like to thank the reviewers and the Editorial staff for the suggestions and recommendations that have greatly contributed to improve this work. We have thoroughly revised the manuscript and answered all queries raised during the reviewing process. Our detailed, point-by-point responses to reviewers’ comments are presented herein.

Reviewer #1:

Thanks for the authors’ updates and revision. The paper is much improved. Some minor comments are attached below:

Page 13 Line 233-239: As for 3.1 Results, I would suggest the authors add a table to describe the sample’s characteristics (e.g., sex, age, level of formal education, income, welfare benefits, household size, oral health literacy, other associated factors, and oral health outcomes), so that readers can better understand the findings for the context and the target population.

Response: In this revised version of the manuscript, we included a table with data related to sample characteristics and associated factors. In addition, we changed the first paragraph of the Results section so there is no redundancy in the data displayed in Table 1 and the text:

*“Sociodemographic characteristics of the sample and descriptive statistics of associated factors are shown in Table 1. Overall, the sample was mostly composed of female participants, individuals with low levels of formal education, recipients of welfare benefits, and users of the Aboriginal community-controlled health centre.”*

**Table 1. Sample characteristics**

| **Variable** | **Frequency** |
| --- | --- |
| **Sociodemographics** |  |
| Sex |  |
| Female | 269 (67.2%) |
| Male | 131 (32.8%) |
| Schooling |  |
| None | 14 (3.5%) |
| Primary school | 34 (8.5%) |
| High school | 257 (64.3%) |
| Technical education | 75 (18.7%) |
| University | 20 (5%) |
| Income |  |
| Job | 89 (22.3%) |
| Welfare | 291 (72.7%) |
| Other | 20 (5%) |
| Welfare |  |
| Health care card | 54 (13.5%) |
| Pension card | 189 (45.3%) |
| Other | 146 (36.5%) |
| None | 11 (2.7%) |
| Age | 34 (24-46)* |
| Household size | 4 (3-6)* |
| **Associated factors** |  |
| Sense of personal control † | 27 (23-32)* |
| Oral health-related self-efficacy | 12 (4-18)* |
| Perceived stress | 28 (26-31)*, |
| Oral health literacy | 47 (40-51)* |
| Oral health-related quality of life | 17.5 (8-28)* |
| **Community engagement** |  |
| Community groups (Yes) | 54 (13.5%) |
| Sporting activities (Yes) | 62 (15.5%) |
| Aboriginal health centre (Yes) | 255 (63.8%) |
| *Median and interquartile interval |  |

.

Page 19 line 370-384: Thanks for the authors’ detailed information on the Australian indigenous context. This context is helpful, but I cannot see how the current study’s findings are interpreted within this context. Are your findings aligned with previous literature you cited? Please discuss based on your findings.

Response: We presented a brief discussion on how our findings can be interpreted within the context of Indigenous Australians:

*“(…) Our study demonstrated that in this rural-dwelling Indigenous Australian community, oral health literacy constitutes a network with other individual and contextual factors. These findings suggest that oral health interventions should consider adopting holistic approaches, beyond exclusively focusing on the transmission of health-specific knowledge. Previous research has documented the limitations of health education strategies to improve health outcomes of Indigenous populations (48, 49). While researchers and Indigenous communities have collaboratively developed culturally appropriate health programs, addressing social determinants of health and socio-economic disadvantage linked to oral health literacy is far more challenging and requires consistent collective and political efforts (50).”*

*“(…) This is particularly relevant for our study as our sample was predominantly composed by young adult women and relatively large families. In Indigenous Australian cultures, the core family unit is often composed by extended family members. In these contexts, child care may be shared by relatives such as mothers, grandparents, siblings, uncles, and aunts (56). Thus, addressing the family as the unit of oral health literacy strategies is essential not only to improve the oral conditions of adults but also to guarantee better health outcomes for the future generations of Indigenous Australians.”*
